# Plasmonic nanogap enhanced phase-change devices with dual electrical-optical functionality

**DOI:** 10.1126/sciadv.aaw2687

**Published:** 2019-11-29

**Authors:** Nikolaos Farmakidis, Nathan Youngblood, Xuan Li, James Tan, Jacob L. Swett, Zengguang Cheng, C. David Wright, Wolfram H. P. Pernice, Harish Bhaskaran

**Affiliations:** 1 Department of Materials, University of Oxford, Parks Road, Oxford OX1 3PH, UK.; 2 Department of Engineering, University of Exeter, Exeter EX4 QF, UK.; 3 Institute of Physics, University of Muenster, Heisenbergstr, 11, 48149 Muenster, Germany.

## Abstract

Modern-day computers rely on electrical signaling for the processing and storage of data, which is bandwidth-limited and power hungry. This fact has long been realized in the communications field, where optical signaling is the norm. However, exploiting optical signaling in computing will require new on-chip devices that work seamlessly in both electrical and optical domains, without the need for repeated electrical-to-optical conversion. Phase-change devices can, in principle, provide such dual electrical-optical operation, but assimilating both functionalities into a single device has so far proved elusive owing to conflicting requirements of size-limited electrical switching and diffraction-limited optical response. Here, we combine plasmonics, photonics, and electronics to deliver an integrated phase-change memory cell that can be electrically or optically switched between binary or multilevel states. Crucially, this device can also be simultaneously read out both optically and electrically, offering a new strategy for merging computing and communications technologies.

## Teaser

Using plasmonics, photonics, and electronics, a nonvolatile memory cell operated both electrically and optically is demonstrated.

## INTRODUCTION

Although integrated photonics has gained great traction over the last decade, primarily for its potential to overcome fundamental limitations of today’s electronic circuitry (*1*), the conversion of electrical and optical signals seamlessly on a chip has remained elusive. The development of compact devices for efficient electro-optic conversion holds great importance as sharing the computing load between the electrical and optical domains shows increasing promise for applications including integrated optical switches, reconfigurable photonic circuits, photonic artificial neural networks, and more (*2*–*4*). Phase-change materials (PCMs) are considered outstanding candidates for dual-mode operation as they, in principle, provide both electrical and optical modulation functionality. To this effect, several devices implementing nonvolatile, optical PCMs have been proposed (*5*, *6*), but none have been successfully demonstrated on an integrated platform. This is because the high electrical contrast between the conductive and insulating state in PCMs requires very close spacing between the metal contacts (usually tens of nanometers) to initiate a phase transition (*7*). In addition, the resulting conductive region formed after electrical switching is, at most, a few hundred nanometers in diameter**—**thus reducing the total volume of material for light-matter interaction (*8*).

Combining plasmonics with PCMs is a particularly promising approach for satisfying such stringent requirements, since the dimensions of such devices can be reduced to tens of nanometers and smaller—significantly below the diffraction limit of traditional optical devices (*9*). The combination of high electrical conductivity and strong plasmonic resonance at optical wavelengths in silver and gold has led to extremely compact electro-optic nanogap devices such as integrated light sources (*10*), photodetectors (*11*, *12*), and modulators (*13*, *14*). In addition, the extremely high field enhancement possible with subwavelength nanogaps enables very high–sensitivity spectral measurements for applications such as label-free detection of biomolecules (*15*, *16*).

While plasmonics permits very strong light-matter interaction in nanometer-scale devices, the relatively high loss of metals at optical frequencies makes guiding light inefficient. Combining integrated photonics with nanoscale plasmonics, however, allows for both low-loss light delivery and strong light-matter interaction in a compact footprint (*17*, *18*). Here, we combine waveguide-integrated plasmonic nanogaps with a PCM, Ge2Sb2Te5 (GST), to create an electro-optic memory cell that is fully addressable in both electrical and optical domains. Previous demonstrations of such mixed-mode devices either have used nonvolatile PCMs, such as VO_2_ (*19*, *20*), which requires significant power consumption for data retention, or have been limited to write/erase operations either electrically or optically but not both (*21*–*24*). By exploiting both the nanoscale dimensions and strong field confinement of a plasmonic nanogap, we enable both electrical and optical nonvolatile switching of GST within the gap, allowing for full mixed-mode operation of a PCM memory cell.

## RESULTS

**Fig. 1:**
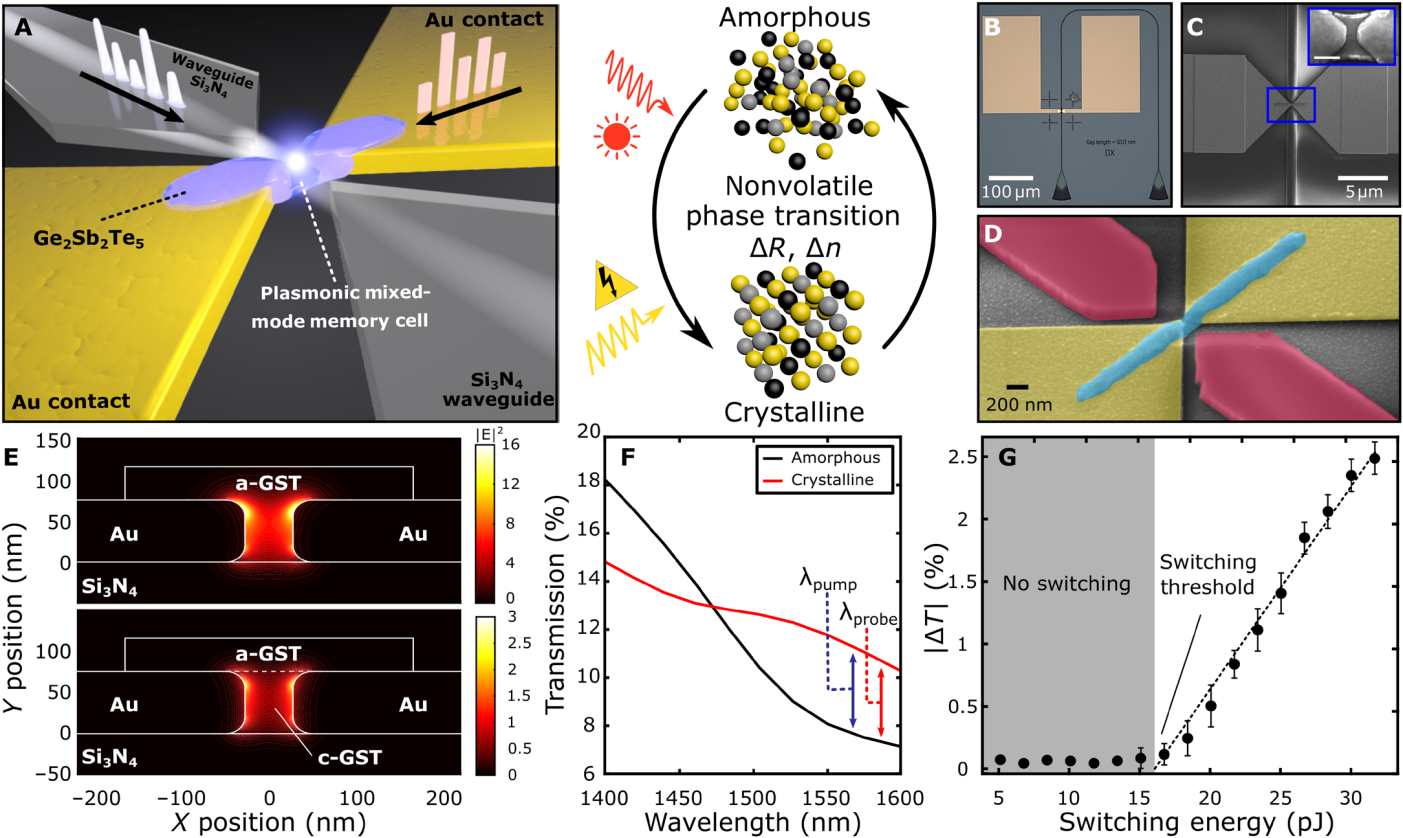
Mixed-mode plasmonic memory cell integrated in a photonic waveguide. (**A**) 3D illustration of device concept. Light is delivered to the nanoscale device via a photonic waveguide, while the Au contacts serve as both device electrodes and plasmonic nanogap to focus incoming light. (**B**) Optical and (**C** and **D**) SEM images of device after fabrication {scale bar [inset of (C)], 100 nm}. The width of the nanogap was measured to be approximately 50 nm for the devices used. (**E**) Eigenmode simulations of field enhancement inside the plasmonic nanogap when the GST is in the amorphous (top) or crystalline state (region between Au electrodes, bottom). The field enhancement is much stronger when GST is in the amorphous state owing to the significantly lower optical loss. (**F**) FDTD simulation of the transmission of device before and after crystallization. The significant change in the refractive index changes the coupling between the nanogap and waveguide, which reduces reflection from the input waveguide, thereby increasing overall transmission of the device in the crystalline state. (**G**) Experimental measurement of total energy in the waveguide required to achieve a nonvolatile phase transition. The switching threshold was measured to be 16 ± 2 pJ according to a linear fit to the data (black dashed line).

A three-dimensional (3D) illustration of our device can be seen in Fig. 1A. We use a partially etched Si_3_N_4_ rib waveguide to route the optical signal to the plasmonic memory cell, which are coupled via a tapered geometry (*25*–*27*). Plasmonic nanogaps are formed between two metal electrodes (3 nm Cr/75 nm Au) fabricated via lift-off using electron-beam lithography (EBL) and thermal evaporation. A thin film (75 nm) of GST with a 5-nm capping layer of SiO_2_ bridges the nanogap, as shown by the atomic force microscope (AFM) micrograph in fig. S1 (see Supplementary Materials), controlling both the electrical resistance and optical transmission of the device depending on the state of the material. By sending either electrical or optical pulses, we can reversibly switch the GST within the nanogap between its highly resistive amorphous phase and conductive crystalline phase (*28*–*30*). Optical and scanning electron microscope (SEM) micrographs of the completed device are shown in Fig. 1 (B to D).

The gold electrodes form a plasmonic metal-dielectric-metal waveguide in the nanogap region that couples to and from the silicon nitride waveguide, allowing for broadband modulation of the optical transmission. The reduction in mode volume serves to both enhance the electric field in the nanogap and reduce the switching volume of the active PCM. To quantify the field enhancement of the plasmonic nanogap, we performed both 2D eigenmode and 3D finite-difference time-domain (FDTD) simulations using Lumerical Solutions and plot the field profile cross sections of the device when GST is in the amorphous and crystalline phases (see Fig. 1E). The amplitude of the electric field intensity is scaled relative to the field amplitude of the waveguide mode. Figure 1E shows that the electric field intensity is enhanced by more than an order of magnitude in the case of amorphous GST due to strong field confinement within the 50-nm nanogap. This enhancement reduces by a factor of 5 (defined by the ratio of maximum field intensity in the nanogap for the amorphous and crystalline states) when the GST within the nanogap switches from a fully amorphous, nonabsorbing state to a fully crystalline, absorptive state. Figure 1F shows the simulated transmission spectrum for the complete waveguide-nanogap system. Here, we observe that, at longer wavelengths, the transmission actually increases when GST in the nanogap is crystalline. This is a result of the modulation of the coupling between the nanogap and waveguide where an increase in the wavelength-dependent refractive index causes an increased coupling to the plasmonic mode within the nanogap. We see in 3D FDTD simulations that the light that is scattered and reflected between the waveguide/nanogap interface decreases when the GST is in the crystalline state. Although the optical absorption also increases for crystalline GST, the enhanced coupling to the nanogap results in an overall increased transmission when GST is switched from the amorphous to the crystalline state.

We experimentally verify this plasmonic field enhancement by sending pulses of increasing amplitude to the device and measuring the nonvolatile change in transmission of a counter-propagating probe signal (λ = 1570 nm). The measured change in transmission as a function of pulse energy can be seen in Fig. 1G. Here, we observe a change in transmission with a pulse energy of 16 ± 2 pJ using a 5-ns optical pulse. Given the linear relationship between switching energy and the change in optical transmission in Fig. 1G, we can explain the modulated transmission of our waveguide-nanogap device as a volume-dependent effect—the volumetric fraction of crystalline domains inside the nanogap grows with increasing pulse energy. We also note that this energy is significantly lower than previous demonstrations of evanescently coupled phase-change devices (*28*) due to the strong field enhancement and small mode volume of our plasmonic nanogap.

**Fig. 2:**
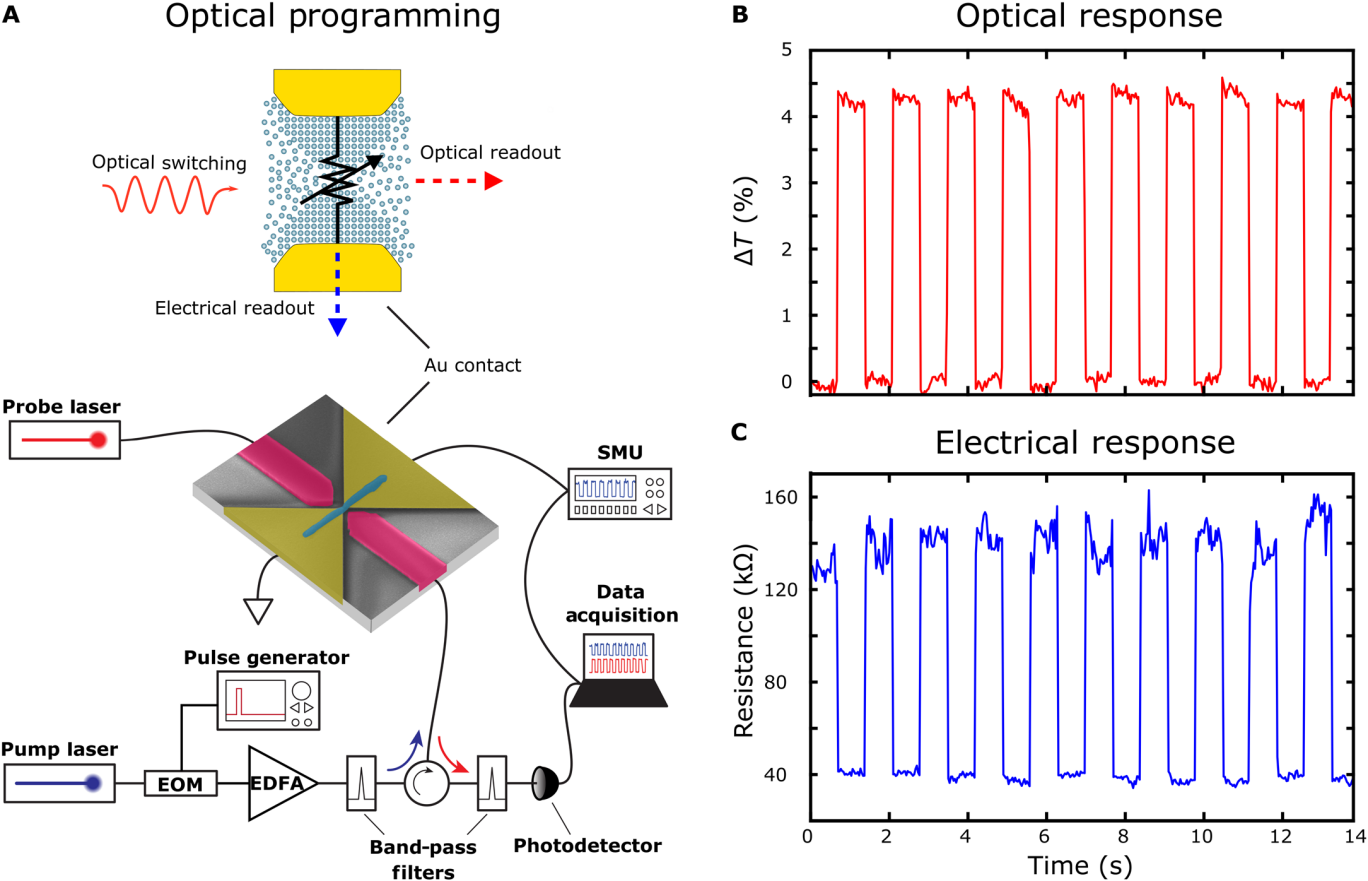
Nonvolatile optical programming of the plasmonic memory cell with electro-optical readout. (**A**) Top: Illustration of device programmed using an optical signal with both electrical and optical readout of the device state. Bottom: Schematic of the experimental setup used for electrical programming of the device. Optical write (piecewise pulse, 7.5 mW for 8 ns *+* 3 mW for 400 ns) and erase (7.5 mW for 8 ns) pulses are used to switch the GST between crystalline and amorphous states, respectively. A continuous wave (CW) optical probe signal and constant voltage source are used to monitor the optical transmission and electrical resistance of the GST simultaneously. Electro-Optic Modulator (EOM), Erbium Doped Fiber Amplifier (EDFA). (**B**) Real-time trace of the device’s optical transmission during multiple write and erase cycles. (**C**) Simultaneous readout of the device’s electrical resistance showing nonvolatile switching of the GST between the amorphous and crystalline states.

We then perform optical programming of our phase-change memory cell as illustrated in Fig. 2A. We send optical write and erase pulses to partially amorphize and crystallize the GST within the nanogap while simultaneously monitoring both the optical transmission and electrical resistance of the device. Figure 2A shows a schematic of the experimental setup where the pump laser is used to switch the GST inside the nanogap. Piecewise optical write pulses (7.5 mW for 8 ns followed by 3 mW for 400 ns) and rectangular erase pulses (7.5 mW for 8 ns) are used to switch the GST between crystalline and amorphous states, respectively. We use a constant-power optical probe to monitor the change in transmission, while a source-meter unit (SMU) in constant voltage mode (*V*_bias_ = 50 mV) is used to monitor the change in resistance. Time-dependent traces of the simultaneous change in both the transmission and resistance of the device can be seen in Fig. 2 (B and C) during consecutive optical write and erase pulses separated by 1 s. In agreement with our FDTD simulations of Fig. 1F, the resistance and transmission traces change as expected—i.e., an amorphization (erase) pulse results in an increase in electrical resistance and decrease in optical transmission, while a crystallization (write) pulse results in the opposite effect.

**Fig. 3:**
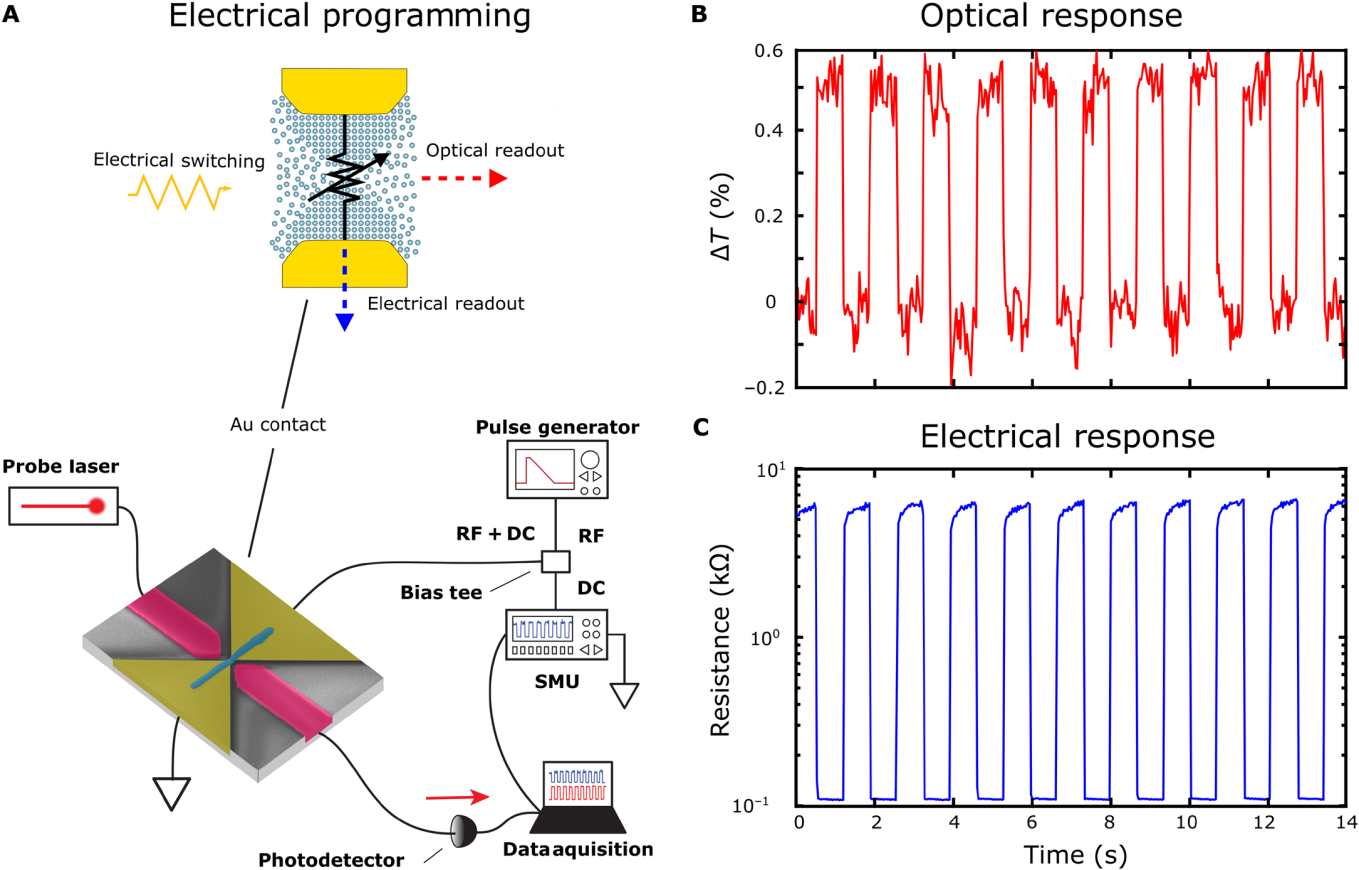
Nonvolatile electrical programming of the plasmonic memory with electro-optical readout. (**A**) Top: Illustration of programming the memory cell using an electrical signal with simultaneous electrical and optical readout. Bottom: Schematic of the experimental setup used for electrically programming the device. Electrical write (rectangular: 350 mV for 10 ns) and erase (triangular: 350 mV with 5-ns/500-ns rise-fall time) pulses are used to switch the state of the GST between the amorphous and crystalline states. Again, a CW optical probe signal and constant voltage source are used to monitor the transmission and resistance simultaneously. (**B**) Real-time trace of the device’s optical transmission during multiple write and erase cycles. (**C**) Simultaneous readout of the device’s electrical resistance showing nonvolatile switching of the GST between the amorphous and crystalline states.

**Fig. 4:**
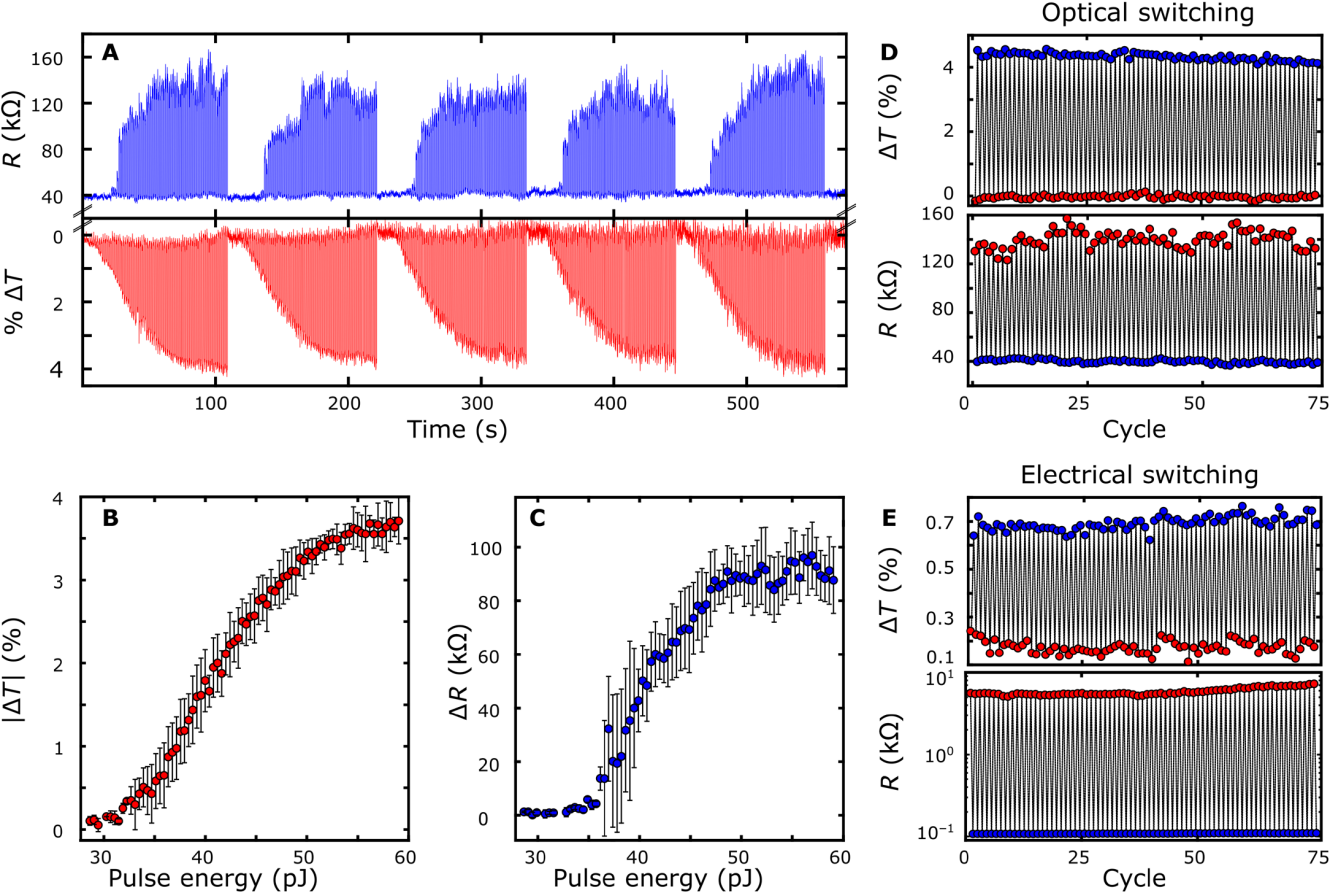
Multilevel operation and cyclability of the mixed-mode device. (**A**) Five consecutive cycles of multilevel operation with a fixed write pulse and a linearly increasing erase pulse energy (8-ns pulse width, 80 erase pulses per cycle). The variation in resistance is much greater than that in optical transmission because of the stochastic nature of crystal domain growth within the nanogap. Cumulative plots of change in (**B**) resistance and (**C**) optical transmission for multilevel traces shown in (A). Cyclability plots of both the electrical resistance and optical transmission during multiple (**D**) optical and (**E**) electrical write and erase cycles.

We subsequently demonstrate successful operation of the device in the electro-optic domain, wherein a change in optical transmission is observed as a result of electrical switching of the device. Here, we add a bias tee between the device and SMU to monitor the DC resistance of the device while sending write and erase pulses via the RF port of the bias tee (see Fig. 3A). By sending a 10-ns, 350-mV pulse across the device (5-ns rise and fall time), we amorphize the GST in the gap, while a 350-mV triangular pulse (5-ns rise, 500-ns fall time) recrystallizes the GST. As shown in Fig. 2 (B and C), the state of the device can be seen in both the optical transmission and electrical resistance readout. As we observed previously and as expected from our FDTD models, the transmission increases when the GST is switched to the crystalline state (see Fig. 2B) and is repeatable for many cycles, as shown in Fig. 4 (D and E). However, in these measurements, we observe higher contrast in the electrical resistance than during optical switching. This is because we are able to switch only the volume of material needed to create or disrupt a conductive path between the electrodes (*31*). The enhanced level of optical sensitivity to such a small volume of material switching between its amorphous and crystalline phase is attributable to the strong light-matter interaction within the plasmonic nanogap. Moreover, higher sensitivity is observed in the optical readout when programming the device optically, whereas higher sensitivity is observed in the electrical reading when the device is programmed electrically. This is attributed to the different energy distribution in the active region of the device when pulsed electrically versus optically. Each mode results in switching at locations of high field strength for that mode. However, this is not the case for the reading of the complementary mode. Crucially, the voltage required to switch the state of the GST is aided by reducing the spacing between the metal contacts and thereby increasing the electric field within the nanogap (see fig. S2). Our device therefore benefits both the optical and electrical design by improving light-matter interaction for the former and reducing the volume and separation between the electrical contacts for the latter, resulting in an efficient mixed-mode device.

As the optical transmission and electrical conductance are both dependent on the fractional volume of crystalline domains, both are dependent on the energy of the write and erase pulses. Figure 4 (A to C) shows the dependence of both the optical transmission and electrical resistance of our device for various optical erase pulse energies (fixed 8-ns pulse width). Between each erase pulse, a fixed piecewise write pulse (1.3 nJ total energy and 408 ns in duration) is used to return the device to the crystalline state. We note that due to the stochastic nature of the formation of amorphous and crystalline domains within the memory cell, the resistance trace of Fig. 4A shows more variation than the readout of the optical probe during optical switching cycles. On the other hand, the variation of the optical transmission is largely limited by the signal-to-noise ratio of our optical readout that can be addressed by increasing the coupling efficiency between the waveguide and plasmonic nanogap, which, based on previous work, could be improved by a factor of 3 (*27*). We attribute this to the different mechanisms between electrical conductance and optical transmission in our device. While the change in optical transmission depends on the overlap between the optical mode and the fractional volume of crystalline versus amorphous domains, a change in electrical conductance requires a continuous path of crystalline domains across the device to be formed—similar to a percolation network (*32*). This results in a dependence on both the fractional volume of crystalline GST and the position of those domains, which leads to greater variation of the device resistance as seen in Fig. 4A. Because of this, the electrical domain both has a higher switching energy threshold and saturates at a lower erase pulse energy than the optical transmission readout, as shown in Fig. 4 (B and C). The lower saturation threshold is due to the fact that once the circuit is fully broken by an erase pulse, greater pulse energies will not affect the overall conductance of the device.

Our devices show good cyclability in both the electrical resistance and transmission for optically and electrically induced switching between the amorphous and crystalline states (see Fig. 4, D and E). The optical and electrical properties of GST and related phase-change chalcogenides have both been demonstrated commercially to be robust for more than 10^12^ write-erase cycles (*7*, *33*). The cyclability of these phase-change chalcogenides, combined with a storage lifetime of more than 10 years at room temperature (*34*), makes our approach highly promising for future integrated electro-optical storage. Table 1 provides a comparison of our work with other nonvolatile photonic memories published to date. Not only do our results compare favorably with the literature in terms of active area and minimum switching energy, but we also show full optical and electrical programming and readout in an integrated device.

**Table 1:** Comparison of various mixed-mode, integrated photonic memory cells. Our work not only significantly reduces the active area of the device but also allows full write-erase programming and readout in both the optical and electrical domains.

**Device**	**Active area (μm^2^)**	**Min. switch** **energy (pJ)**	**Mixed-mode** **programming?**	**Mixed-mode** **readout?**	**Nonvolatile?**	**References**
VO_2_ on waveguide	2.0 × 4.0	1.4 × 10^3^	No	Yes	No	(*19*)
VO_2_ on waveguide	0.35 × 0.5	9 × 10^2^	Yes	Yes	No	(*20*)
Germanium Telluride Nanowire (GeTe-NW) on waveguide	0.25 × 1.0	8 × 10^3^	No	Yes	Yes	(*21*)
GST on Surface Plasmon Polariton (SPP) waveguide	0.5 × 2.0	6.9 × 10^3^	Yes	No	Yes	(*22*)
GST on waveguide	1.0 × 1.3	67 ± 3	No	No	Yes	(*28*)
GST plasmonic nanogap	0.05 × 0.05	16 ± 2	Yes	Yes	Yes	This work

## DISCUSSION

We have demonstrated a nonvolatile nanoscale electro-optic device that enables both electrical and optical programming and readout using the synergetic combination of PCMs and nanoplasmonics. This is an unprecedented demonstration of an integrated, reversible, and nonvolatile phase-change memory cell that fully bridges the gap between electro-optic mixed-mode operations. This was enabled by using a plasmonic design that simultaneously reduces the footprint of the device, enhances light-matter interaction, and reduces the separation between electrical contacts, creating a compact and highly sensitive device. Our approach also enables a direct comparison of both optical and electrical read and write operations in a single device, demonstrating the relative merits and limitations of both. The nonvolatile nature of our platform provides an exciting outlook in the development of switchable and reconfigurable metadevices by means of optical or electrical stimuli, enabling novel approaches to switchable metamaterial-based optical components (*35*–*37*). We anticipate that a plethora of novel devices and platforms should arise in the coming years, which will capitalize on the bridge between the electrical and photonic domains that are demonstrated here. These devices potentially herald true device-level integration of hybrid optoelectronic computing platforms with in-memory computing and multilevel data storage, which is readily applicable to this work (*38*, *39*).

## MATERIALS AND METHODS

### Sample fabrication

The mixed-mode nanogap devices were fabricated on silicon wafers containing a plasma-enhanced chemical vapor deposition 330-nm Si_3_N_4_ top layer on a 3.43-μm SiO_2_ buried oxide. A JEOL JBX-5500ZD 50-kV EBL system was used to write the photonic circuitry using the MaN-2403 negative tone resist. Subsequently, reactive ion etching in CHF_3_/Ar/O_2_ was carried out to etch 165 nm of the Si_3_N_4_ and thus obtain the photonic devices. A second EBL step on the CSAR 62 positive tone resist was used to define windows for metal deposition via thermal evaporation. Last, a third EBL step in the CSAR 62 resist was implemented to define apertures for GST-SiO_2_ deposition. A stack of 75 nm of GST with a 10-nm silicon dioxide oxide capping layer (to avoid oxidation) was deposited in an argon environment using a homebuilt RF sputtering system (Nordiko).

### Measurement setup

The optoelectronic experimental setups shown in Figs. 2 and 3 were used to carry out the time-resolved, mixed-mode, and multilevel measurements. For optical programming, a tunable laser source (N7711A, Keysight) was used as the seed, which was modulated with an electro-optical modulator (2623NA, Lucent Technologies) controlled by a 100-MHz electrical pulse generator (AFG3102C, Tektronix) to generate optical pulses. These optical pulses were further amplified using a low-noise erbium-doped fiber amplifier (AEDFA-CL-23, Amonics). To avoid interference with the probe laser (TSL-550, Santec), two different wavelengths were used: 1560 and 1570 nm for the pump and probe, respectively. Both the pump pulses and the probe were coupled into the photonic device using integrated grating couplers with a coupling efficiency of ~20%. Circulators and optical filters (OTF-320, Santec) were used to separate the probe from the pump signal for monitoring the transmission level. A 200-kHz low-noise photoreceiver (New Focus, 2011) was used to measure the optical transmission over time. Together with the optical probe, a Keithley 2614B source meter unit was used to simultaneously monitor the electrical resistance state of the GST during optical programming.

For electrical programming, the 100-MHz electrical pulse generator was connected to the nanogap electrodes through the RF port of a bias tee (ZFBT-4R2GW+, Mini-Circuits) to supply electrical pulses, while the resistance state was monitored using the DC port and the Keithley SMU. The optical probe transmission was simultaneously recorded with the electrical resistance state of the GST using the 200-kHz low-noise photoreceiver as before.

## Supplementary Material

http://advances.sciencemag.org/cgi/content/full/5/11/eaaw2687/DC1

Download PDF

Plasmonic nanogap enhanced phase-change devices with dual electrical-optical functionality

## SUPPLEMENTARY MATERIALS

Supplementary material for this article is available at http://advances.sciencemag.org/cgi/content/full/5/11/eaaw2687/DC1 Section S1. Mixed-mode device architecture Section S2. Topography measurements of the full mixed-mode device and cross section of the GST bridge Section S3. Electrical switching threshold dependence Section S4. Optical properties of GST Section S5. Comparison of switching and readout mechanisms in the mixed-mode device Section S6. Additional FDTD simulations for partially crystallized GST Section S7. Multilevel electrical and optical programming Section S8. Photosensitivity of the phase-change memory Fig. S1. Detailed description of the device architecture. Fig. S2. AFM scans of the device focusing on the active region of the PCM. Fig. S3. Voltage threshold requirement for electrical switching of the PCM. Fig. S4. Optical constants (*n* and *k*) of GST used in the FDTD simulations. Fig. S5. Illustration for understanding switching and readout mechanism in mixed-mode nanogap devices. Fig. S6. Simulation results for four different crystallization conditions in the mixed-mode nanogap. Fig. S7. Multilevel electrical and optical programming versus programming energy. Fig. S8. Photoconductive effect of the device in amorphous state.
