# Supplementary material for: Plasmonic nanogap enhanced phase-change devices with dual electrical-optical functionality
Source: Sci Adv. 2019 Nov 29;5(11):eaaw2687. doi: 10.1126/sciadv.aaw2687 (PMC6884412; doi:10.1126/sciadv.aaw2687)
Supplement: http://advances.sciencemag.org/cgi/content/full/5/11/eaaw2687/DC1 [file supp_5_11_eaaw2687__index.html]

Science Advances | Science AdvancesAAASSearchScience AdvancesMenu

## Supplementary Materials

**This PDF file includes:**

- Section S1. Mixed-mode device architecture
- Section S2. Topography measurements of the full mixed-mode device and cross section of the GST bridge
- Section S3. Electrical switching threshold dependence
- Section S4. Optical properties of GST
- Section S5. Comparison of switching and readout mechanisms in the mixed-mode device
- Section S6. Additional FDTD simulations for partially crystallized GST
- Section S7. Multilevel electrical and optical programming
- Section S8. Photosensitivity of the phase-change memory
- Fig. S1. Detailed description of the device architecture.
- Fig. S2. AFM scans of the device focusing on the active region of the PCM.
- Fig. S3. Voltage threshold requirement for electrical switching of the PCM.
- Fig. S4. Optical constants (*n* and *k*) of GST used in the FDTD simulations.
- Fig. S5. Illustration for understanding switching and readout mechanism in mixed-mode nanogap devices.
- Fig. S6. Simulation results for four different crystallization conditions in the mixed-mode nanogap.
- Fig. S7. Multilevel electrical and optical programming versus programming energy.
- Fig. S8. Photoconductive effect of the device in amorphous state.

Download PDF

**Files in this Data Supplement:**

- Adobe PDF - aaw2687\_SM.pdf
