# Supplementary material for: Plasmonic nanogap enhanced phase-change devices with dual electrical-optical functionality
Source: Sci Adv. 2019 Nov 29;5(11):eaaw2687. doi: 10.1126/sciadv.aaw2687 (PMC6884412; doi:10.1126/sciadv.aaw2687)
Supplement: Download PDF [file aaw2687_SM.pdf]

Science Advances
AAAS

advances.sciencemag.org/cgi/content/full/5/11/eaaw2687/DC1

Supplementary Materials for

Plasmonic nanogap enhanced phase-change devices with dual electrical-optical functionality

Nikolaos Farmakidis*, Nathan Youngblood*, Xuan Li, James Tan, Jacob L. Swett, Zengguang Cheng, C. David Wright, Wolfram H. P. Pernice, Harish Bhaskaran*

*Corresponding author. Email: harish.bhaskaran@materials.ox.ac.uk

Published 29 November 2019, *Sci. Adv.* **5**, eaaw2687 (2019)
DOI: 10.1126/sciadv.aaw2687

This PDF file includes:

Section S1. Mixed-mode device architecture
Section S2. Topography measurements of the full mixed-mode device and cross section of the GST bridge
Section S3. Electrical switching threshold dependence
Section S4. Optical properties of GST
Section S5. Comparison of switching and readout mechanisms in the mixed-mode device
Section S6. Additional FDTD simulations for partially crystallized GST
Section S7. Multilevel electrical and optical programming
Section S8. Photosensitivity of the phase-change memory
Fig. S1. Detailed description of the device architecture.
Fig. S2. AFM scans of the device focusing on the active region of the PCM.
Fig. S3. Voltage threshold requirement for electrical switching of the PCM.
Fig. S4. Optical constants (n and k) of GST used in the FDTD simulations.
Fig. S5. Illustration for understanding switching and readout mechanism in mixed-mode nanogap devices.
Fig. S6. Simulation results for four different crystallization conditions in the mixed-mode nanogap.
Fig. S7. Multilevel electrical and optical programming versus programming energy.
Fig. S8. Photoconductive effect of the device in amorphous state.

Section S1. Mixed-mode device architecture

In order to efficiently couple the optical mode from the waveguide into the plasmonic metal slot, a tapered region is used in the waveguide leading up to the gold electrodes. The precise geometry of the tapered region of the device as well as the plasmonic metal slot are detailed in fig. S1. Top-view as well as cross-sectional view schematics of the device prior to the phase change element deposition are illustrated in fig. S1B and C. A scanning electron micrograph of the complete device post deposition is seen in fig. S1A. A complete description of the phase change element deposited inside the metal slot is discussed in detail in section S2.

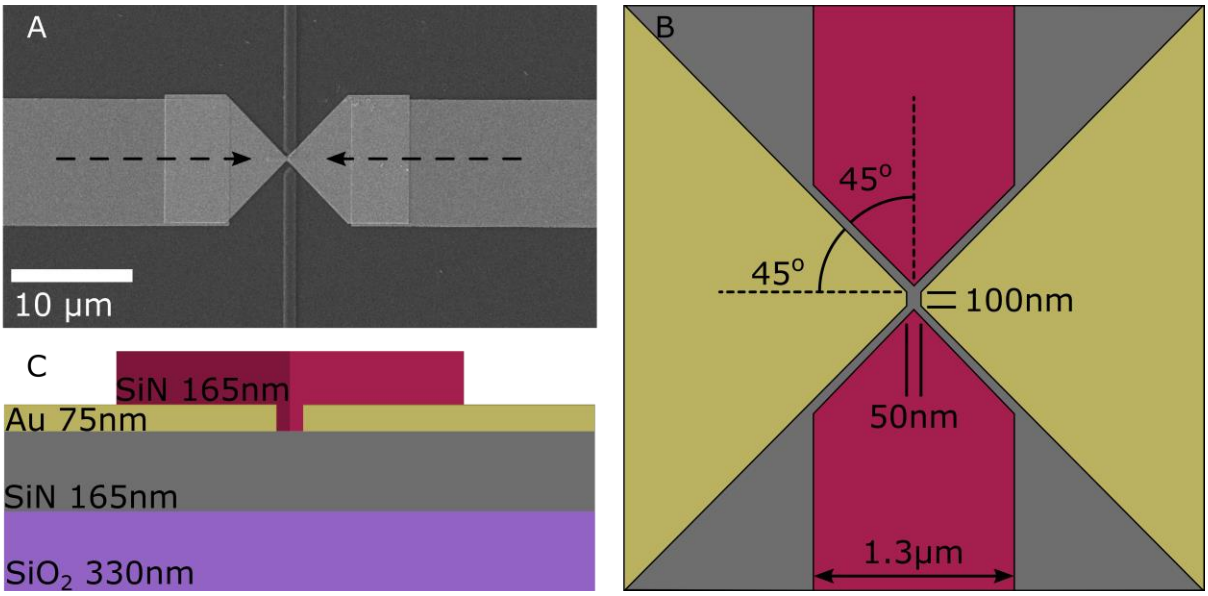

Fig. S1. Detailed description of the device architecture. (**A**) Scanning electron micrograph of the complete device. Arrows indicate the section drawn in (C). (**B**) Top-view, dimensioned schematic of the plasmonic mode converter. (**C**) Cross-sectional view of the device indicating the individual stacks that make up the mixed-mode cell.

Section S2. Topography measurements of the full mixed-mode device and cross section of the GST bridge

In order to evaluate the exact topography of our mixed mode device, we collect atomic force microscope (AFM) micrographs and focus on the active region of the device and in particular the GST bridge. The micrographs complement our SEM measurements and demonstrate that the PCM has indeed been deposited inside the slot between the gold electrodes. A topography cross-section of the $Ge_2Sb_2Te_5$ bridge is shown in fig. S1A where a decrease in height is evident between the electrodes after deposition. Depositing the PCM between the electrodes rather than bridging the electrodes in a semi-suspended geometry, allows for high sensitivity in the optical mode and makes up a robust structure as evident by the cyclability of the device.

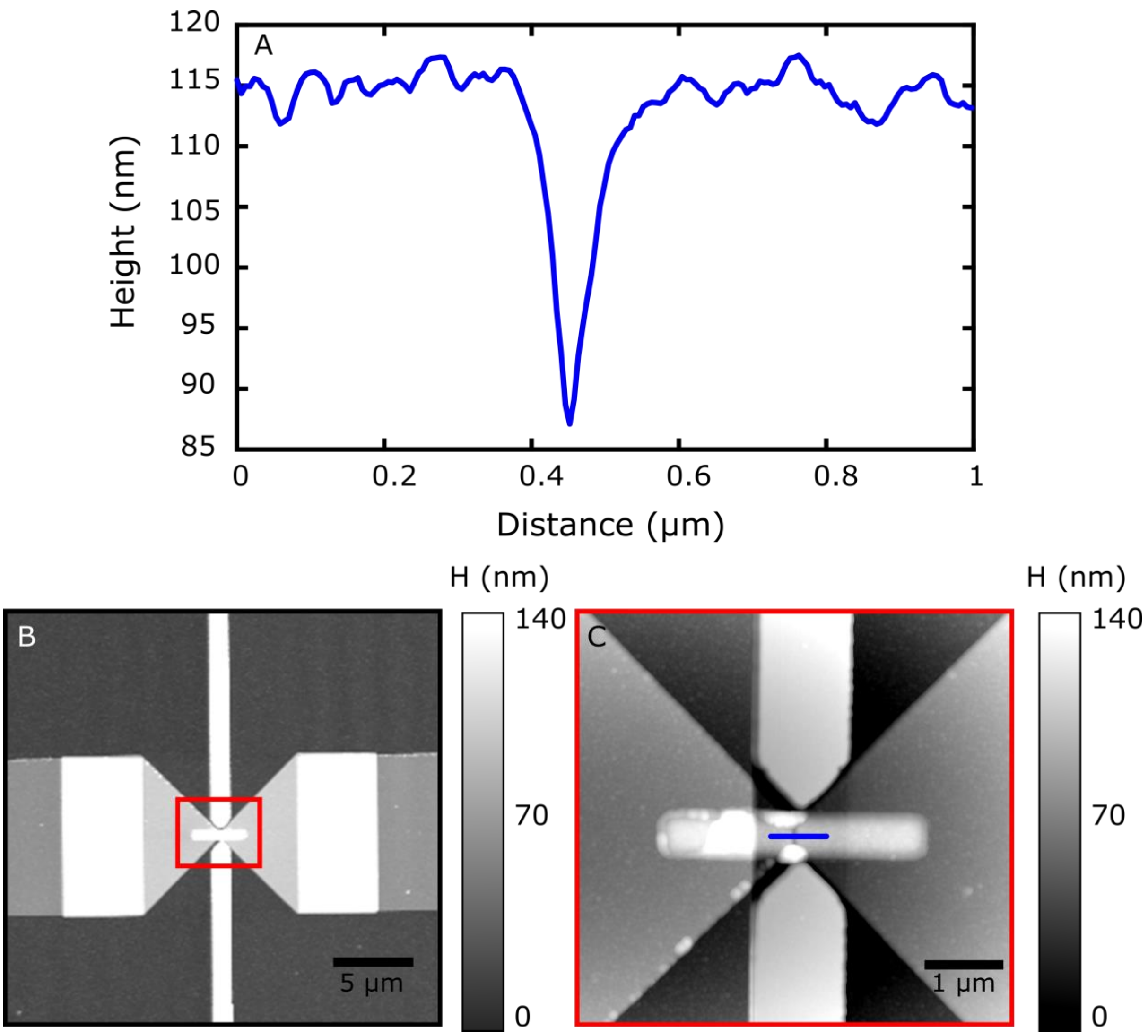

Fig. S2. AFM scans of the device focusing on the active region of the PCM. (**A**) Section plot of the region highlighted in figure showing a decrease in height between the electrodes. (**B**) and (**C**) Topography micrographs of the waveguide, electrodes and PCM. The PCM inside the nanogap as opposed to the less efficient bridged geometry.

Section S3. Electrical switching threshold dependence

As expected, it was found that the voltage required to switch the PCM increased as the distance between the gold electrodes is increased. In the devices demonstrated, we use a 50nm gap between the electrodes which allows us to switch the material with less than 1V. Such low switching thresholds make our devices highly compatible with standard computing elements and could be readily integrated as electronic memory cells.

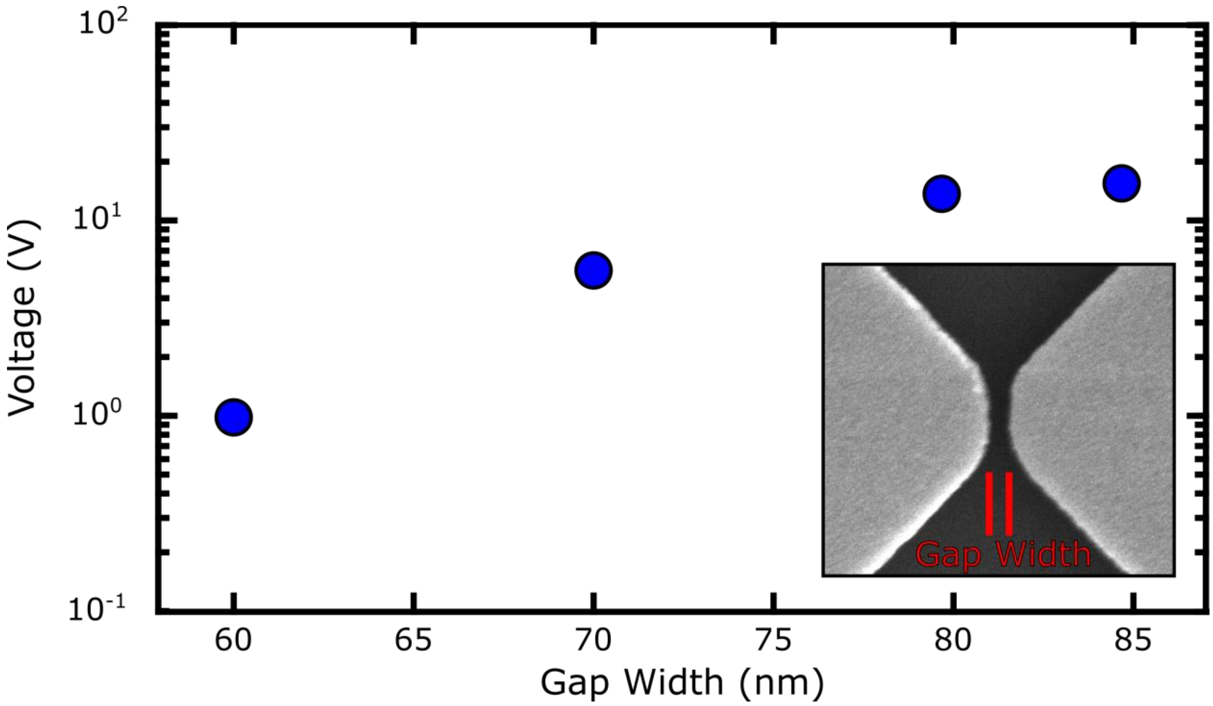

Fig. S3. Voltage threshold requirement for electrical switching of the PCM. As expected, the switching threshold increases as the distance between electrodes increases. We achieve sub 1V switching thresholds for nanogaps with separation widths smaller than 60nm. The results shown were collected by performing standard IV ramp curves with increasing amplitude until switching occurred.

Section S4. Optical properties of GST

The optical properties of the PCM cell were derived by sputtering 50nm $Ge_2Sb_2Te_5$ on a silicon substrate (base pressure of 2E-6 torr, 30W RF power, and 5 mtorr working pressure) and subsequently performing ellipsometry measurements to obtain the properties of the amorphous phase. The samples were then switched to the crystalline state by heating to 200^0C for 10 minutes and re-measured to determine the properties of the crystalline phase. The optical constants of the integrated PCM cell were assumed to maintain the optical properties of the measured thin films. The measured optical constants were used in the Lumerical Solutions® FDTD and MODE simulations and are shown in fig. S4.

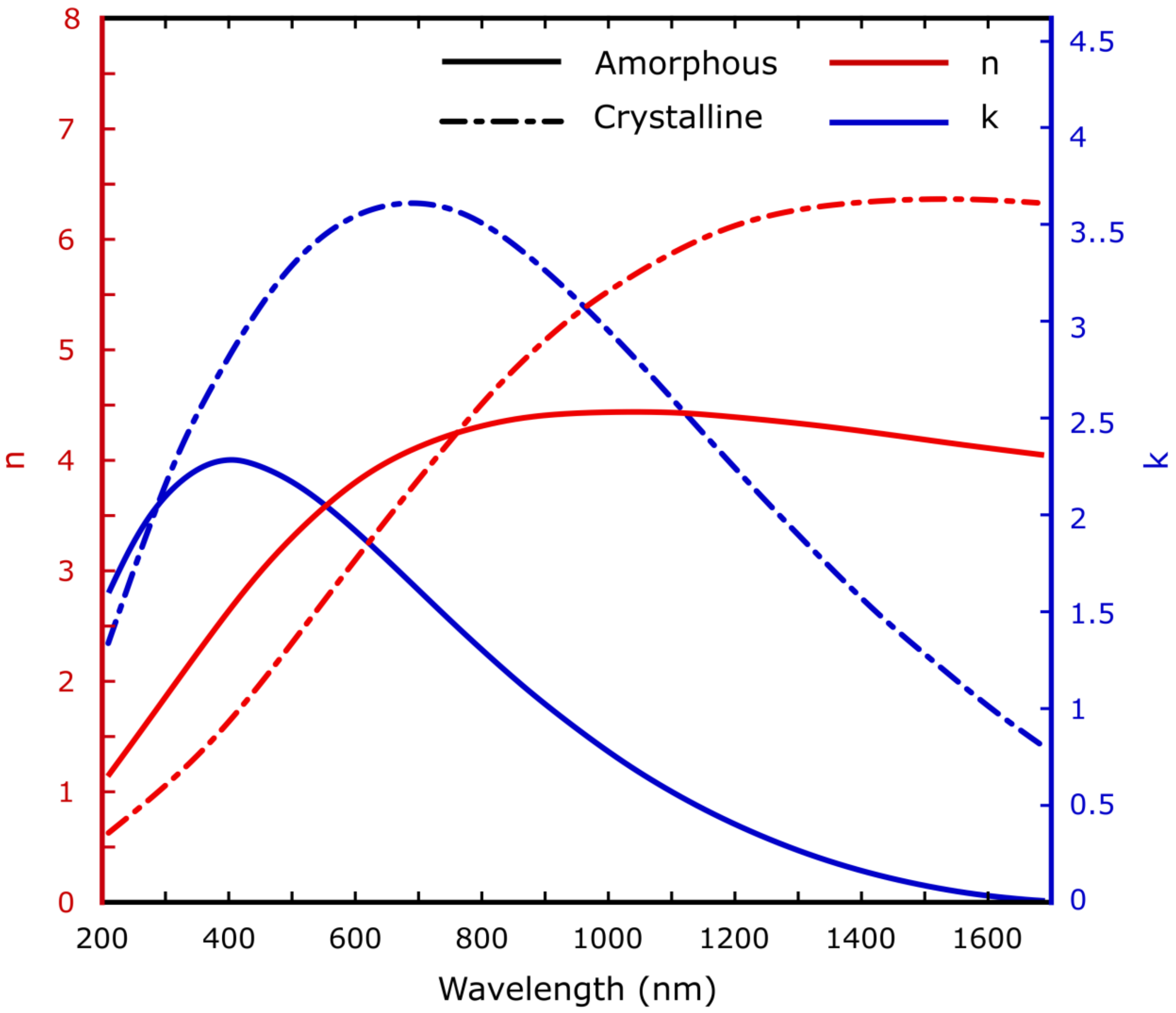

Fig. S4. Optical constants (*n* and *k*) of GST used in the FDTD simulations.

Section S5. Comparison of switching and readout mechanisms in the mixed-mode device

The difference in resistance between Fig. 2 and 3 of the main text can be explained by the different switching and readout mechanisms between the electrical and optical domain (illustrated in fig. S5). Because optical readout of the device depends on the total volume of amorphous versus crystalline GST in the nanogap, combined with the optical mode overlap with these respective phases of GST, there can be large optical contrast in a device with relatively high electrical resistance. When the device is switched optically and measured electrically as in Fig. 2, crystalline domains are randomly created within the amorphous GST in the nanogap where the optical mode intensity is highest and do not necessarily create a low resistance electrical path between the two electrodes (fig. S5B). This can be seen in the variable and high-resistance electrical readout of Fig. 2C.

For the case of electrically switched GST in Fig. 3, we see a much lower device resistance. This is due to the field-dependent switching mechanism in GST, where a low resistance path is created between two electrodes through a combination of field-induced threshold switching and current-induced Joule heating (fig. S5A). Since this switching mechanism ensures a conductive path is formed (or broken) between the two electrodes, the device resistance is much lower and less variable in the case of electrical switching. This fundamental difference between electrical and optical switching of phase-change materials is clearly differentiated in our nanoscale device for the first time which allows simultaneous optical and electrical readout under different switching mechanisms.

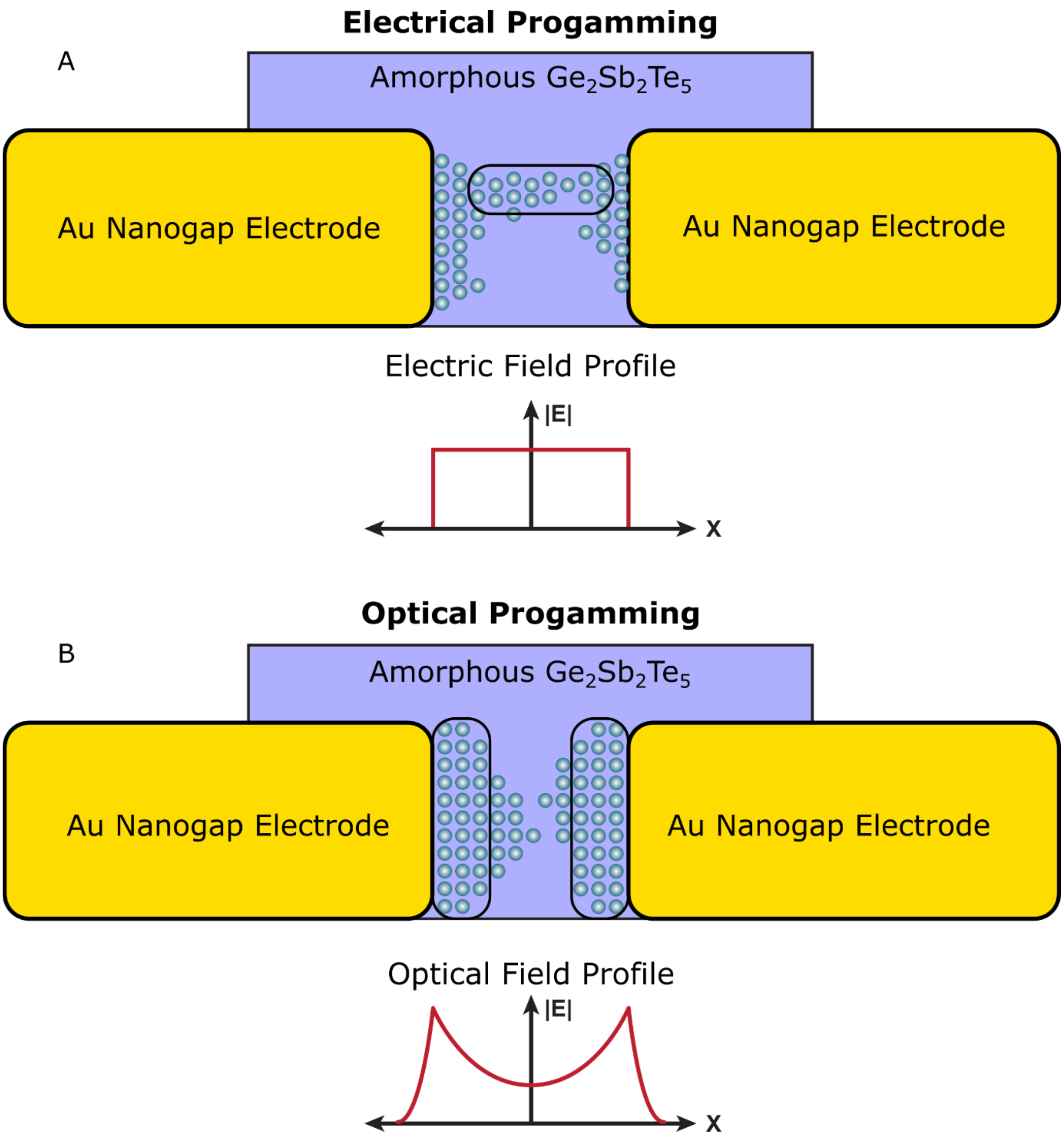

Fig. S5. Illustration for understanding switching and readout mechanism in mixed-mode nanogap devices. (**A**) In the case of electrical programming, a conductive path between the two electrodes forms and is broken in the highest resistance region (white circled region) due to both threshold switching and Joule heating of the GST. (**B**) For optical programming, the area in the nanogap that has the largest overlap with the plasmonic waveguide mode experiences the highest absorption (white circled regions) and therefore switches due to optical heating of the GST. Note: for the case of optical programming, a conductive path is not necessarily formed between the two electrodes.

Section S6. Additional FDTD simulations for partially crystallized GST

In order to better understand how the optical transmission varies with different programming modalities, we ran full 3D FDTD simulations for four different crystallization conditions in the nanogap. Figure S6 shows the geometries used to approximate the case where crystalline GST of comparable volume is switched either electrically (fig. S6B) or optically (fig. S6C) as discussed in section S5 of the supplementary. Figure S7 shows the resulting transmission spectra calculated based on the four geometries in fig. S6. One can see good qualitative agreement with the experimental results in Fig. 2 and 3 of the main text where an optically programmed nanogap shows higher optical contrast than an electrically programmed nanogap. Further reducing the dimensions of the nanogap to achieve greater overlap between the optical and electrical modes would further enhance the mixed mode contrast for both regimes.

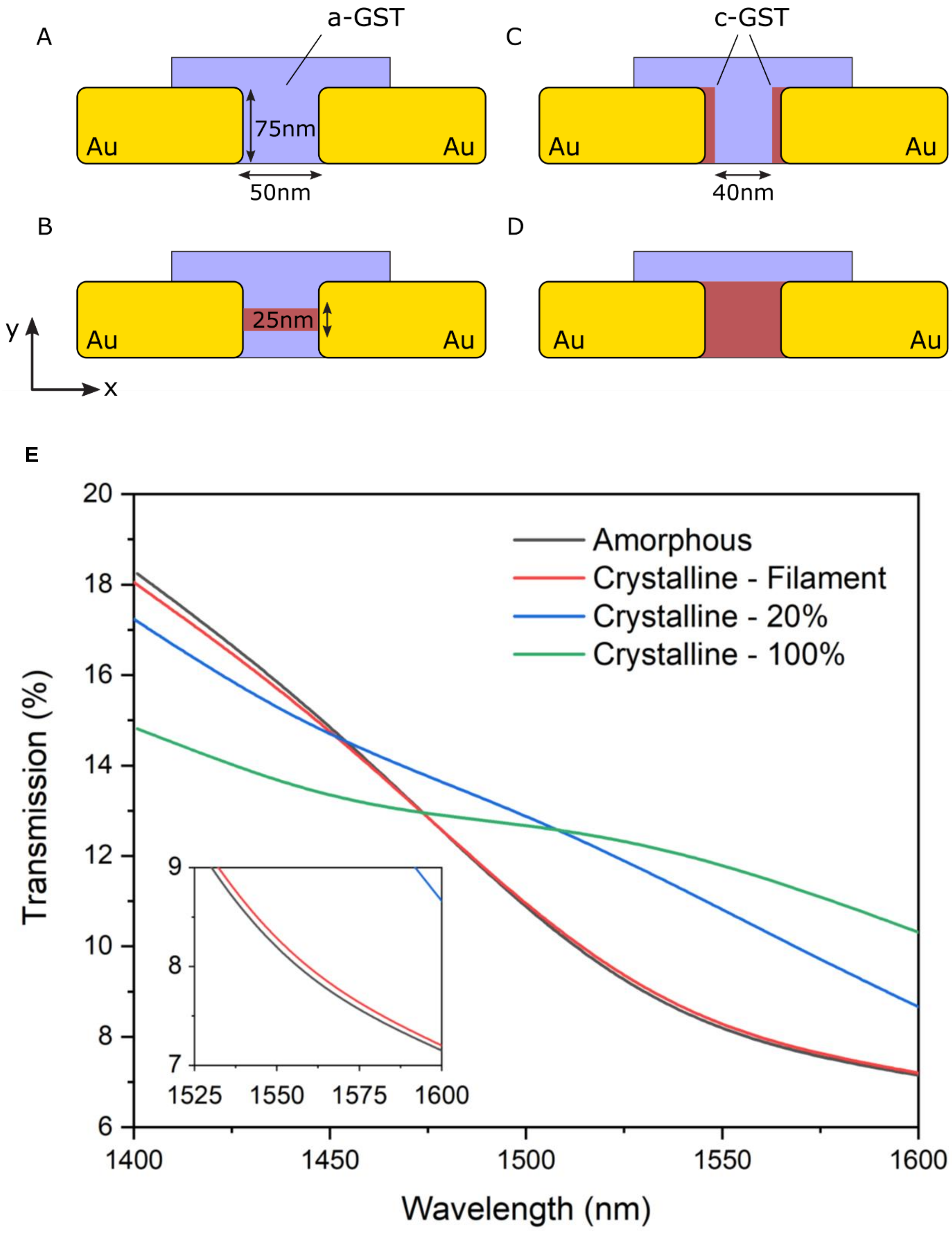

Fig. S6. Simulation results for four different crystallization conditions in the mixed-mode nanogap. (**A**) Fully amorphous, (**B**) electrically switched filament (25nm diameter), (**C**) optically switched partial crystallization (20%), (**D**) optically switched full crystallization (100%). **(E)** Due to the small overlap between the electrically switched filament (25 nm in diameter) and the plasmonic waveguide mode, a much smaller change in transmission is observed for electrically switched GST compared with optically switched GST.

Section S7. Multilevel electrical and optical programming

Here, it is shown that multiple levels of crystallinity can be programmed not only using optical pulses as shown in Fig. 3A-C but also using electrical pulses of different amplitudes. Figure S8A demonstrates the modulation of the conductance when twenty pulses of linearly increasing amplitude are sent between 0 and 0.5V with a 5ns-500ns rise-fall time (crystallization pulse energies ranging from 0pJ to 8.5pJ). Similarly, fig. S8B demonstrates 20 optical pulses of increasing energy between 30pJ and 60pJ. While both electrical and optical pulses are capable of modulating the crystallinity of the device we find that when programmed electrically, the device is sensitive to the formation of the conductive path between the electrodes whereas during optical programming, the device is modulated consistently over a larger range.

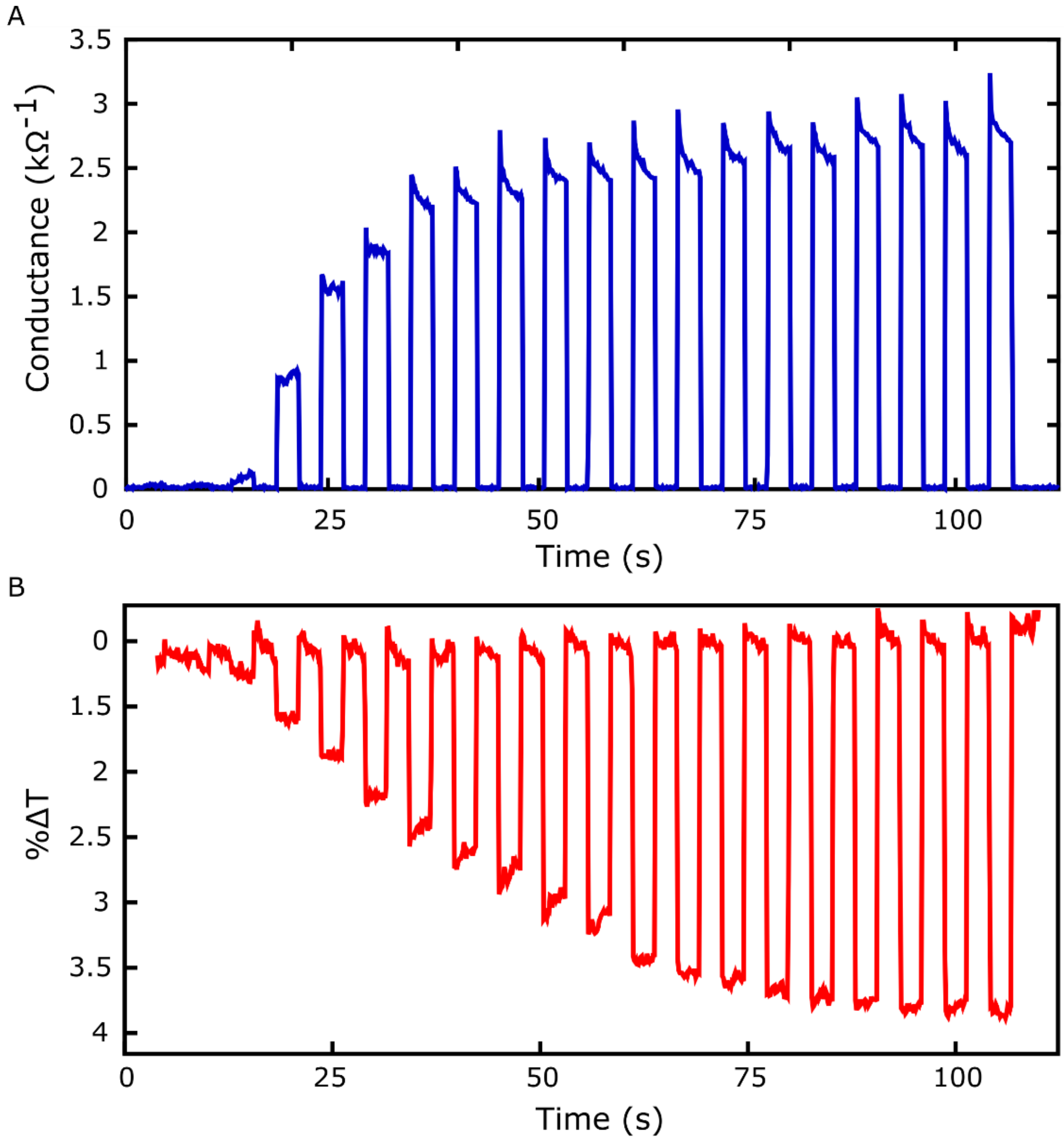

Fig. S7. Multilevel electrical and optical programming versus programming energy. Lower density is achieved using electrical pulses (**A**) compared to optical pulses (**B**). This is attributed to the formation of a conductive path between the electrodes which is insensitive to the formation of other similar paths at a later switching operation. Conversely, when optical pulses are used, crystalline volumes of GST are formed at the metal-dielectric interface thereby modulating the transmission between fully amorphous and fully crystalline.

Section S8. Photosensitivity of the phase-change memory

Here, we observe a volatile response in the conductivity of the phase change element due to optical irradiation. Figure S9 shows the device's photoconductive response at an applied bias of 100 mV collected by the nanoelectrodes while sequentially turning on and off the CW laser. As expected, we observe a linear increase in photocurrent with increasing laser power as well as a low dark current (9 nA) when the laser is off. The ability of the device to simultaneously act as both a non-volatile memory element and a photodetector could enable a plethora of applications for on-chip optoelectronic communication.

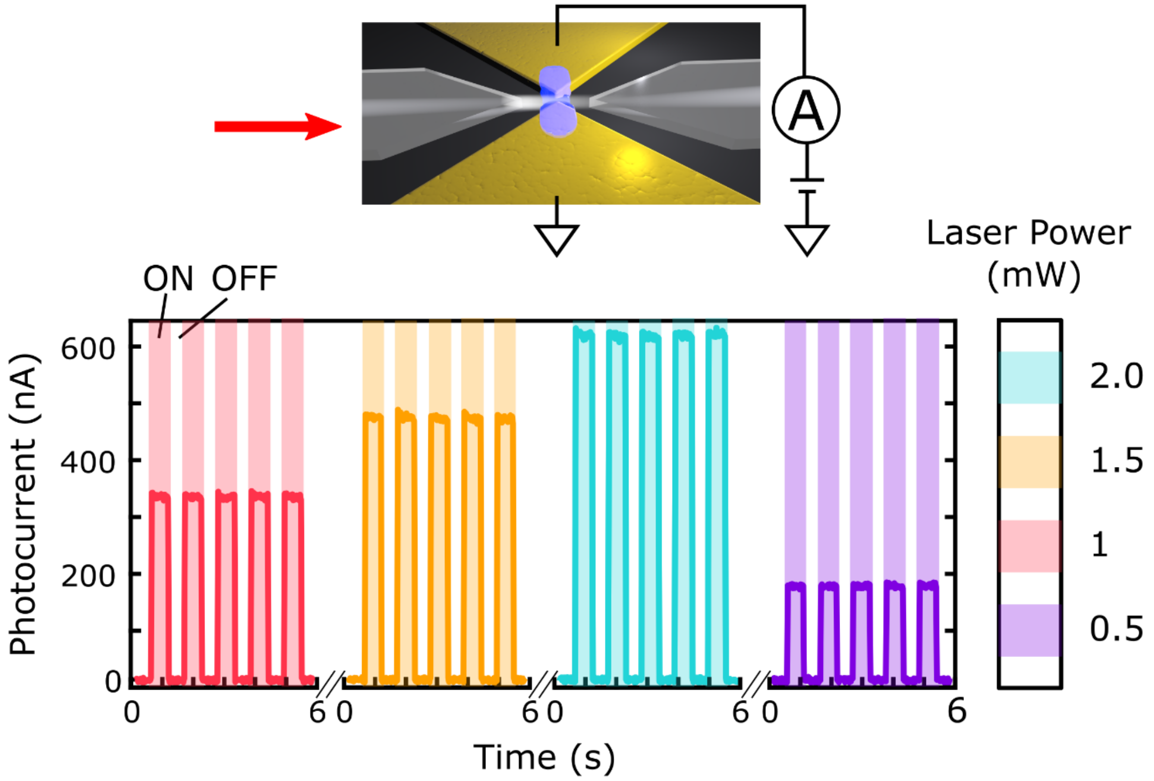

Fig. S8. Photoconductive effect of the device in amorphous state. Photocurrent is observed when the device is biased with a DC voltage (100 mV) due to filling of trap states in the GST. Sensitivity is enhanced due to the strong light-matter interaction in the plasmonic nanogap.
